# Influence of Dulaglutide on Serum Biomarkers of Atherosclerotic Plaque Instability: An Interventional Analysis of Cytokine Profiles in Diabetic Subjects—A Pilot Study

**DOI:** 10.3390/medicina60060908

**Published:** 2024-05-30

**Authors:** Marcin Hachuła, Michał Kosowski, Marcin Basiak, Bogusław Okopień

**Affiliations:** Department of Internal Medicine and Clinical Pharmacology, Medical University of Silesia, Medyków 18, 40-752 Katowice, Poland; marcin.hachula@gmail.com (M.H.); mkosowski@sum.edu.pl (M.K.); bokopien@sum.edu.pl (B.O.)

**Keywords:** GLP-1 receptor agonist, diabetes mellitus, pentraxin 3, MMP-9, copeptin, lipoprotein(a), semaglutide, dulaglutide

## Abstract

*Background and Objectives*: The rise in global diabetes cases, reaching a staggering 529 million in 2021 from 108 million in 1980, underscores the urgency of addressing its complications, notably macrovascular ones like coronary artery, cerebrovascular, and peripheral artery diseases, which contribute to over 50% of diabetes mortality. Atherosclerosis, linked to hyperglycemia-induced endothelial dysfunction, is pivotal in cardiovascular disease development. Cytokines, including pentraxin 3 (PTX3), copeptin, lipoprotein(a) [Lp(a)], and matrix metalloproteinase-9 (MMP-9), influence atherosclerosis progression and plaque vulnerability. Inhibiting atherosclerosis progression is crucial, especially in diabetic individuals. Glucagon-like peptide 1 receptor agonists (GLP-1 RAs), increasingly used for type 2 diabetes, show promise in reducing the cardiovascular risk, sparking interest in their effects on atherogenesis. This study sought to examine the effects of glucagon-like peptide-1 receptor agonists (GLP-1 RAs) on biomarkers that indicate the instability of atherosclerotic plaques. These biomarkers include pentraxin 3 (PTX3), copeptin (CPC), matrix metalloproteinase-9 (MMP-9), and lipoprotein(a) [Lp(a)]. *Materials and Methods*: A total of 34 participants, ranging in age from 41 to 81 years (with an average age of 61), who had been diagnosed with type 2 diabetes mellitus (with a median HbA1c level of 8.8%), dyslipidemia, and verified atherosclerosis using B-mode ultrasonography, were included in the study. All subjects were eligible to initiate treatment with a GLP-1 RA—dulaglutide. *Results*: Significant reductions in anthropometric parameters, blood pressure, fasting glucose levels, and HbA1c levels were observed posttreatment. Moreover, a notable decrease in biochemical markers associated with atherosclerotic plaque instability, particularly PTX3 and MMP-9 (*p* < 0.001), as well as Lp(a) (*p* < 0.05), was evident following the GLP-1 RA intervention. *Conclusions:* These findings underscore the potential of GLP-1 RAs in mitigating atherosclerosis progression and plaque vulnerability, thus enhancing cardiovascular outcomes in individuals with type 2 diabetes mellitus.

## 1. Introduction

The global diabetic population is on the rise, estimated to have increased from 108 million in 1980 to 529 million in 2021 [1]. People with diabetes are at an increased risk of complications, particularly macrovascular complications such as coronary artery disease, cerebrovascular disease, and peripheral artery disease. These complications are major contributors to mortality, accounting for more than 50% of the deaths of diabetic patients [2]. Atherosclerosis is the primary cause of cardiovascular illnesses [3]. Hyperglycemia is strongly linked to endothelial dysfunction, which not only initiates the formation of atherosclerotic plaques but also contributes to their progression and instability [4]. The pathomechanism of atherosclerosis is highly complex, involving multiple stages, such as initiation due to endothelial dysfunction, inflammatory cell migration, atherosclerotic plaque formation, and eventual plaque rupture. Numerous cytokines play key roles in each of these stages [5]. The clinical implication of atherosclerotic cardiovascular disease (ASCVD) is associated with the vulnerable plaques. This term encompasses different phenotypes of unstable atherosclerotic plaques, such as plaque rupture, intraplaque hemorrhage, erosion, and plaque fissuring [6]. Cytokines involved in the vulnerability of atherosclerotic plaques include, e.g., pentraxin 3 (PTX3), matrix metalloproteinase 9 (MMP9), lipoprotein(a) [Lp(a)] and copeptin (CPC). 

Pentraxin 3 is emerging as a promising immunoinflammatory marker for evaluating cardiovascular risk. Within the pentraxin family, which comprises acute-phase proteins characterized by a pentameric structure, C-reactive protein (CRP) is one of the members. Importantly, while CRP is predominantly synthesized in the liver, PTX3 is locally produced and released by various cell types, particularly monocytes/macrophages. The production of PTX3 is stimulated by pro-inflammatory cytokines such as interleukin 1 (IL-1), tumor necrosis factor α (TNFα), and oxLDL [7]. The distinctive capability of pentraxin 3 to regulate local inflammation involving macrophages and smooth muscle cells has prompted research into its role in the pathogenesis of atherosclerosis and cardiovascular disease. The observed positive correlation between the PTX3 concentration and the risk of adverse outcomes in patients with coronary artery disease (CAD) suggests that PTX3 could be a valuable biomarker for cardiovascular disease [8,9]. Furthermore, the serum PTX3 concentration is correlated with plaque vulnerability evaluated by optical coherence tomography in individuals with coronary artery disease [10,11].

The precursor peptide preprovasopressin produced in the hypothalamus is responsible for the release of arginine vasopressin (AVP) and an equal amount of copeptin. Their secretion occurs, among other factors, in response to stress and affects the regulation of the endocrine response of the hypothalamo–pituitary–adrenal (HPA) axis. The precise function of copeptin remains elusive. In contrast to AVP, measuring serum copeptin concentrations is more accessible. Therefore, its primary role lies in serving as an indirect indicator of the circulating serum levels of AVP [12]. Copeptin could be a promising new marker for diagnosing acute cardiovascular events. Numerous studies indicate that, when evaluated alongside cardiac troponin (cTn), copeptin is effective in swiftly ruling out myocardial infarction. Furthermore, in cases of stroke, myocardial infarction, or heart failure, it can be employed for risk stratification and prognosis assessments [13].

Lp(a) is an independent risk factor for ASCVD [14]. Lipoprotein(a) plays a role in various stages of atherosclerosis. Within the endothelium, it undergoes more pronounced oxidation than LDL, and thus intensifies the action of adhesion molecules. Lp(a) increases the synthesis of other pro-inflammatory cytokines such as IL-1, IL-6, and TNFα. Lipoprotein(a) is also involved in the instability of atherosclerotic plaques. Its prothrombotic and antifibrinolytic effects contribute to intravascular thrombotic processes [15,16,17]. In recent years, a correlation has been established between elevated serum lipoprotein(a) levels and the presence of vulnerable atherosclerotic plaques in patients experiencing acute cardiovascular events [18,19].

Matrix metalloproteinases (MMPs) are enzymes that play a vital role in modifying the extracellular matrix and assisting in the recruitment of white blood cells to areas of inflammation. As a result, they serve as important controllers of the inflammatory process. Excessive or unbalanced release of MMP-9 is associated with tissue damage in inflammation [20]. MMP-9 contributes to the progression of arteriosclerosis. Numerous studies indicate a correlation between elevated serum levels of matrix metalloproteinase-9 and increased risks of plaque rupture and acute cardiovascular events [21].

Hence, inhibiting the progression of atherosclerosis and preventing its instability has huge clinical significance in enhancing the prognosis of individuals with diabetes. In the treatment of type 2 diabetes, there is an increase use of ‘new hypoglycemic drugs’, such as glucagon-like peptide 1 receptor agonists (GLP-1 RAs). These medications, besides affecting blood glucose levels through pleiotropic effects, also influence various cardiac risk factors [22]. In recent years, several randomized clinical trials of GLP-1 RAs have demonstrated a substantial reduction in cardiovascular risk [23,24,25,26]. The mechanism behind this phenomenon remains incomprehensible. In connection with the decrease in cardiovascular events among patients diagnosed with ASCVD, there was a hypothesis that these drugs affect the atherogenesis [27].

Consequently, the objective of our study was to explore the impact of dulaglutide on biomarkers of atherosclerotic plaque instability, including PTX3, MMP-9, Lp(a), and CPC.

## 2. Materials and Methods

### 2.1. Study Population

**Figure 1:**
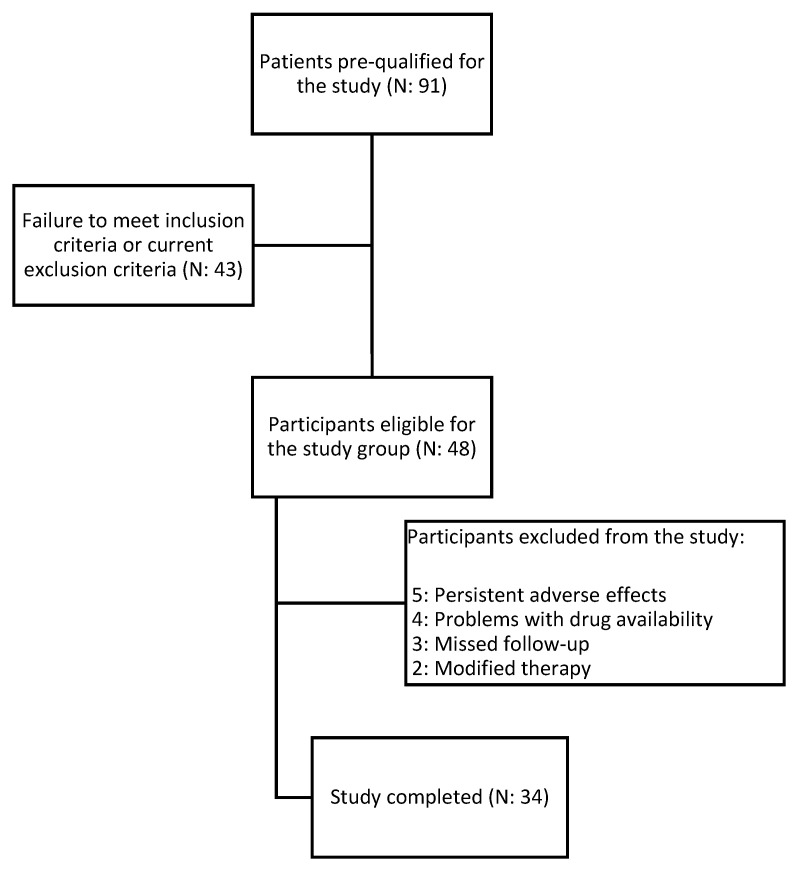
Flowchart of the study.

We selected 34 participants, aged 41–81 years (mean: 61 years), from a total of 91 patients for the study. All participants were diagnosed with type 2 diabetes mellitus, dyslipidemia, and verified atherosclerosis based on B-mode ultrasonography of the carotid artery. Only those who met strict and specific criteria for inclusion and exclusion were deemed qualified to take part in the study. Participants were enrolled at the Department of Internal Medicine and Clinical Pharmacology in Katowice, Poland, as well as through recommendations from the Mysłowice and Imielin diabetes outpatient departments. The study protocol received approval from the Bioethical Committee of the Medical University of Silesia under reference number PCN/CBN/0052/KB1/45/I/22. Informed permission was obtained from every patient in compliance with the Declaration of Helsinki. The data pertaining to the subjects were anonymized. The medical experiment took place from January 2022 to September 2023. Participants received dulaglutide at a typical hypoglycemic dose. Treatment was initiated with a dose of 1.5 mg. Patients who did not achieve significant glycemic control after 4 weeks had their dose increased to 3 mg, provided they tolerated adverse events. The drug was administered every week at the same time of day. During the observation, other medications were not modified. The intervention lasted for a duration of 180 days. Figure 1 shows the flowchart of the study.

### 2.2. Inclusion and Exclusion Criteria

The criteria for inclusion were as follows: individuals with type 2 diabetes and dyslipidemia, which was defined as having a blood total cholesterol (TC) level greater than 190 mg/dL and/or triglyceride (TG) level greater than 150 mg/dL or were receiving statin therapy for previously diagnosed dyslipidemia. All study participants also were required to have confirmed atherosclerotic plaques on a carotid ultrasound and acceptance of dulaglutide therapy. 

Patients were excluded from the study in the following cases: type 1 diabetes with confirmed antibodies, acute cardiovascular conditions such as unstable coronary artery disease, exacerbation of chronic heart failure, and a history of percutaneous coronary intervention (PCI), coronary artery bypass grafting (CABG), or stroke within 3 months prior to study initiation. Furthermore, exclusion criteria included acute and chronic pancreatitis, acute exacerbation of autoimmune disorders, abnormal levels of thyrotropin hormone, and any symptomatic acute infection defined as an increase in CRP values > 5 mg/dL or leukocytosis. Additionally, patients with a glomerular filtration rate (eGFR) below 45 mL/min/1.73 m^2^, classified as chronic kidney disease (CKD) stage G3b; acute and chronic liver diseases (indicated by transaminase levels exceeding 3 times the norm); or a medical history of diagnosed chronic viral hepatitis were also excluded. Failure to provide informed consent, alcohol dependency, pregnancy, and breastfeeding were also criteria for exclusion from the study. After 6 months, at the end-of-intervention visit, the medical history was reviewed again. Patients were excluded if they had increased physical activity, modified their diet under the guidance of a nutritionist, initiated new medications known to affect cardiovascular health (such as fibrates, ezetimibe, niacin, non-selective beta-blockers, metformin, sodium-glucose transporter 2 (SGLT2) inhibitors, ursodeoxycholic acid, angiotensin-converting enzyme inhibitors or angiotensin receptor blockers, or direct oral anticoagulants = DOACs), or had their therapy modified due to an exacerbation of chronic conditions within the last 6 months. Additionally, exclusion criteria were applied if patients experienced a coronary or stroke incident or suffered a severe infection. 

### 2.3. Laboratory and Anthropometric Measurements

All measurements were taken before study enrollment and after 6 months of treatment by a physician. Standard procedures were used to assess body weight and height, and body mass index (BMI) was computed as kilograms per square meter (kg/m^2^). Measurements of waist and hip circumferences were taken at the standard anatomical sites, and the waist-to-hip ratio (WHR) was calculated. The arterial blood pressure (BP) was assessed on two occasions while the individual was seated, utilizing the Omron M400 Intelli IT, Frosinone, Italy, automated equipment. The eGFR was estimated using the CKD-EPI formula and the results were reported in mL/min/1.73 m^2^. Standard laboratory measurements were conducted in the accredited laboratory, and blood samples were taken from the veins after a 12 h fasting period at 8 a.m., both before and after 180 days of treatment. A blood sample was collected to test for the presence of a blood clot, and it was promptly placed in a centrifuge for separation. The serum levels of cytokines were assessed using the following commercially available enzyme-linked immunosorbent assay (ELISA) kits, as described by the manufacturers: Lp(a) (10-1106-01, Mercodia AB, Uppsala, Sweden); active form of MMP-9 (SEA553Hu; Cloud-Clone Corporation, Houston, TX, USA); CPC (CEA365HU; Cloud-Clone Corporation, Houston, TX, USA); and active form of PTX3 (RD191477200R, BioVendor, Brno, Czech Republic). For Lp(a), the values were converted from U/L to mg/dL with the conversion rate provided by the manufacturer.

### 2.4. Arteriosclerotic Plaque Examination

The extracranial region of the carotid arteries underwent an examination using B-mode and color Doppler ultrasonography, utilizing a linear probe operating at a frequency of 7.5–10 MHz on a Hitachi Aloka F37, Osaka, Japan ultrasound machine. Confirmation of atherosclerotic plaque presence in the carotid artery was determined by a C-IMT complex thickness exceeding 1.5 mm or the observation of atherosclerotic plaque.

### 2.5. Statistical Analysis

The data were analyzed using Statistica TIBCO Software Inc., Palo Alto, CA, USA (2017) version 13.3 software, which was legally obtained by the Medical University of Silesia in Katowice. We employed the Shapiro–Wilk test to evaluate the normality of distributions. The values are reported as either means and 95% confidence intervals or medians with Q1–Q3 values. The *t*-test for independent means and *t*-test for dependent means were employed to compare quantitative variables. The Mann–Whitney U test was employed to compare independent variables with a non-normal distribution. We employed the Wilcoxon test to analyze the dependent variables. We considered a *p*-value below 0.05 to be statistically significant.

## 3. Results

### 3.1. Study Group Characteristics

The study group consisted of 36 patients with a mean age of 61 ± 10.4 years. Women accounted for 56% (*n* = 19) of the study group. The mean BMI was 36.29 ± 6.9, and 76% participants were obese (BMI > 30 kg/m^2^). The average WHR was 0.978 ± 0.065. All subjects were diagnosed with type 2 diabetes mellitus (median glycated hemoglobin (HbA1c) level: 8.8%; average duration of 10.4 years) and dyslipidemia. The concomitant diseases included hypertension (82%), chronic kidney disease (24%—all in stage G3a), hypothyroidism (18% of everyone with a normalized thyroid-stimulating hormone (TSH) value) and heart failure with a reduced ejection fraction (11%). For diabetes treatment, all patients chronically received metformin, sulfonylurea was used by 44% participants, along with sodium-glucosium like transporter 2 inhibitors (20%), dipeptidyl peptidase 4 (DPP-4) inhibitors (3%) and insulin (26%). The most common treatments for comorbidities were HMG-CoA reductase inhibitors (88%), fibrates (18%), and angiotensin converting enzyme inhibitors or angiotensin receptor blockers (82%). At baseline, median serum levels of total cholesterol were 165.5 mg/mL, low-density lipoprotein-cholesterol (LDL-C) were 86 mg/dL, high-density lipoprotein-cholesterol (HDL-C) were 48.45 mg/dL and triglycerides (TG) were 169.5 mg/dL. Median levels of alanine transaminase (ALT) were 27 U/L and mean aspartate transaminase (AST) levels were 33.2 ± 14.3 U/L, respectively, and the median systolic blood pressure (SBP) was 135.5 mmHg. There were 17% active smokers, and none of the patients abused alcohol. In total, 12 subjects (35%) met the World Health Organization (WHO) criteria for physical activity. Detailed data are presented in Table 1. 

### 3.2. Biochemical Effect after Treatment

In the study group, we obtained statistically significant reduction in anthropometric parameters after treatment, including BMI (*p* < 0.001); on average, patients lost 5.25 kg of weight. Substantial differences in decreased blood pressure for SBP (*p* < 0.001) and DPB (*p* < 0.001) were noted. Lower concentrations of fasting glucose and an average HbA1c reduction of 1.03% (mean: 7.77%) (*p* < 0.001) were observed. Eleven (32%) individuals achieved the target value for HbA1c. Regarding the lipid profile, changes in the LDL fraction, TGs, non-HDL cholesterol, and HDL fractions did not reach statistical significance. Furthermore, we obtained a statistically significant decrease in the fibrosis-4 score (FIB-4) (*p* < 0.001). Detailed results are presented in Table 2. 

### 3.3. Effect of Treatment on Biochemical Markers of Atherosclerotic Plaque Vulnerability

Following a 180-day administration of GLP-1 RAs in our trial group, we noticed a significant reduction in biochemical markers linked to the fragility of atherosclerotic plaques, including PTX3, MMP-9 (*p* < 0.001), and Lp(a) (*p* < 0.05). In terms of CPC, despite achieving a decrease in its concentration, the change was not statistically significant. The results are presented in Table 3.

### 3.4. Occurrence of Adverse Events

Five patients did not complete the study due to the severity of the adverse effects; among the study group, 21 patients reported experiencing adverse effects. They mostly concerned the gastrointestinal system: 26% reported nausea, 26% reported diarrhea, and 20% reported a feeling of fullness. No serious adverse events were reported during the study. Only 14% of patients described the above-mentioned side effects as impairing normal functioning. 

## 4. Discussion

Our study demonstrates the beneficial effects of GLP-1 receptor agonists on various factors increasing the cardiovascular risk in patients with type 2 diabetes (T2D). We assessed anthropometric and biochemical parameters, as well as the serum concentrations of cytokines known to influence plaque vulnerability, in individuals treated with GLP-1 receptor agonists before and after treatment.

As type 2 diabetes progresses, arteries become increasingly vulnerable to atherosclerosis development and the onset of cardiovascular complications, such as myocardial infarction [28]. Large randomized clinical trials (RCT) of GLP-1 RAs, including PIONEER, REWIND, SUSTAIN and LEADER, have shown their positive effects on reducing the cardiovascular risk [23,24,25,26]. The mechanism behind this action remains unexplained and is the focus of numerous ongoing studies worldwide. Our hypothesis posited that GLP-1 receptor agonists influence various stages of atherogenesis. Recently published papers have illustrated that GLP-1 RA therapy reduces the concentrations of proinflammatory cytokines responsible for initiating atherosclerotic plaques [29]. In this study, our objective was to examine the impact of treatment with a GLP-1 receptor agonist on the process of atherosclerotic plaque stabilization by assessing serum markers indicative of plaque vulnerability.

Over the past few years, pentraxin 3, which actively participates in various stages of atherosclerosis development, has gained attention among researchers as a new biochemical marker for the cardiovascular risk [9]. Numerous studies have shown that increased PTX3 levels are associated with the presence of high-risk plaques [10,11,30] and acute cardiovascular events [8,31,32,33,34]. In our work, a therapeutic intervention with dulaglutide resulted in a decrease in serum PTX3 levels in patients diagnosed with diabetes and dyslipidemia. In the available literature, there are reports of a positive effect of liraglutide on reducing the expression of PTX3 in primary human epithelial cells taken from the umbilical vein [35]. In animal model studies, the administration of exenatide reduced serum PTX3 levels in rats [36]. In the same human study conducted by Suzuki et al., a rise in PTX3 levels was noted among patients following liraglutide treatment [37]. Discrepancies in the findings could be attributed to the inclusion of participants with parallel dyslipidemia and ultrasound-confirmed atherosclerosis in our study group. Notably, the study by Suzuki et al. lacked information about cardiovascular disease in their participant cohort. Pentraxin 3 and its heightened levels are linked with atherosclerotic cardiovascular disease [9].

The role of MMP-9 in atherosclerotic plaque instability is well understood and widely studied. There are numerous reports showing that serum MMP-9 levels may be a prognostic factor for future adverse cardiovascular events [38]. Our 180-day treatment intervention involving GLP-1 receptor agonists led to a statistically significant reduction in serum matrix metalloproteinase 9 levels in patients with diabetes and dyslipidemia. The findings of our study are consistent with the results of previous research conducted on animal models [39,40,41,42]. As far as we know, there have been no studies comparing the effects of GLP-1 receptor agonists on MMP-9 levels in patients at high risk of cardiovascular disease.

Anything that disrupts the body’s homeostatic balance, such as an acute cardiovascular incident, as well as the erosion of atherosclerotic plaques, activates the HPA axis, leading to an increase in concentrations of stress hormones. One of the major hypothalamic stress hormones stimulated by various stressors is vasopressin (AVP), along with copeptin, which are released in an equimolar ratio [43]. As a biomarker, copeptin has been well studied in cardiovascular disease, obtaining very satisfying results [13,44]. In our study, we observed a reduction in copeptin levels among patients who were treated with GLP-1 Ras; however, these changes were not statistically significant. In their work, Svenja Leibnitz and colleagues obtained a statistical decrease in copeptin levels in healthy volunteers and in patients with polydipsia treated with dulaglutide [45]. On the other hand, in healthy volunteers, intervention with another analog—exenatide—was not associated with a significant decrease in copeptin levels; it is worth noting that the blood drawn for the study came from six people [46]. Treatment with liraglutide had no statistically significant effect on copeptin levels in women with polycystic ovary syndrome (PCOS) [47]. It is essential to consider that GLP-1 RAs may reduce water intake, potentially influencing copeptin concentrations in the organism [48]. The findings from our study contribute to the investigation of copeptin’s potential role as a biomarker for cardiovascular risk. Based on our current understanding, there are no comparative studies clarifying the influence of GLP-1 RAs on copeptin levels in diabetic patients.

In patients with coronary artery disease, a link between increased lipoprotein(a) levels and atherosclerotic plaque instability has been proven [18,19,49,50]. Several large RCTs, including JUPITER, LIPID and AIM-HIGH, have found that Lp(a) remains a predictor of CVD events, even in patients treated with potent statins who achieve low LDL concentrations [51,52]. Therefore, it is important in modern therapy to use drugs with a pleiotropic mechanism, which will further reduce the cardiovascular risk by, e.g., decreasing lipoprotein(a) levels. In our study group, therapy with GLP-1 RAs resulted in a significant decrease in lipoprotein(a) levels. It is worth noting that we did not observe a significant difference in typical lipidogram fractions such as LDL or non-HDL values. Similar results to ours were obtained by Bechlioulis A et al.; in patients with type 2 diabetes treated with liraglutide, he obtained an improvement in Lp(a) values [53]. Similar observations in a diabetes cohort were made by Steven R et al. [54]. On the other hand, Ariel D et al. did not observe a change in lipoprotein(a) concentration in patients with an overdiabetic state during liraglutide therapy [55]. There is a significant need for large-scale cross-sectional studies to definitively assess changes in lipoprotein(a) levels during treatment with GLP-1 RAs, given the high baseline cardiovascular risk in patients with diabetes and the multi-pronged benefit of using these drug groups.

Interestingly, in our cohort, changes in the lipid profile did not reach statistical significance. Moreover, the mean values for LDL and non-HDL for the whole group after treatment were slightly higher. This may be due to several factors. Firstly, patients in our study group had a preexisting diagnosis of dyslipidemia and were undergoing treatment with statins (88% of individuals); at baseline, 42% of patients had target LDL cholesterol levels, and 47% had target triglyceride levels. Following therapy, these percentages increased to 59%. Secondly, after a careful analysis of each case, a few patients experienced an increase in LDL and non-HDL values by about 50 mg/dL, resulting in an increase in the median value. This increase is not statistically significant. If we excluded these patients, we would observe a significant decrease in the median values.

Our study is subject to various limitations. One disadvantage of this study is the absence of a control group that received either a placebo or an active comparator. Secondly, our study group is a relatively small sample size of only 34 patients. Finally, it is important to note that this study exclusively focused on patients from the Upper Silesia region of Poland, and the outcomes may vary based on factors such as residence, race, and environmental conditions.

## 5. Conclusions

To sum up, in addition to the already known positive effect of GLP-1RAs on the metabolic and anthropometric parameters of diabetic patients treated with them, these drugs significantly reduce the concentrations of PTX3, MMP-9, and Lp(a), which are involved in plaque vulnerability. This effect could be responsible for reducing the cardiovascular risk in patients with diabetes. Additional studies, especially randomized clinical trials, are needed to indisputably assess the impacts of these drugs on the development of cardiovascular diseases and the risk of cardiovascular events.

## Figures and Tables

**Table 1:** Baseline characteristics of patients. BMI—body mass index; WHO—World Health Organization.

	Study Group
Number of patients, n	34
Age, years	61
Women, *n* (%)	19 (56%)
Men, *n* (%)	15 (44%)
Body mass, kg	102.6
Height, cm	168
BMI, kg/m^2^	36.29
Overweight, *n* (%)	8 (24%)
Obese, *n* (%)	26 (76%)
WHO guidelines on physical activity, n (%)	12 (35%)
Smokers, *n* (%)	
Active	6 (17%)
Past	9 (26%)
Alcohol abuse, %	0
Comorbidity, *n* (%)	
Hypertension	28 (82%)
Ischemic heart disease	10 (29%)
Chronic kidney diseases	8 (24%)
Thyroid diseases	6 (18%)
Heart failure	4 (11%)

**Table 2:** Comparison of the biochemical effect of dulaglutide between the study groups before and after the intervention. ALT—alanine transaminase; AST—aspartate transaminase; BMI—body mass index; DBP—diastolic blood pressure; FIB-4—fibrosis-4 score; GFR—glomerular filtration rate; GGTP—gamma-glutamyl transpeptidase; HbA1C—glycated hemoglobin; HDL—high-density lipoprotein cholesterol; LDL—low-density lipoprotein cholesterol; SBP—systolic blood pressure; TGs—triglycerides; TC—total cholesterol; WHR—waist/hip ratio; SD—standard deviation; Q1—first quartile; Q3—third quartile.

	Study Group before Treatment			Study Group after Treatment			
	Mean	SD		Mean	SD	*p* Value	
**BMI kg/m^2^**	36.29	6.9		34.4	7	<0.001	
**HbA1C %**	8.8 *	1.3		7.77	1.04	<0.001	
**GFR mL/min/1.73 m^2^**	68.09	12.4		70.09	18.28	0.44	
**Weight kg**	102.61	22.4		97.37	22.8	<0.001	
**WHR**	0.98	0.065		0.97	0.06	0.42	
**SBP mmHg**	135.53	13.05		129.5	9.57	<0.01	
**Creatinine mg/dL**	1.09	0.15		1.07	0.18	0.6	
**ASP U/I**	33.21	14.3		29.47	10.3	0.14	
	**Median**	**Q1**	**Q3**	**Median**	**Q1**	**Q3**	***p*** **Value**
**DBP mmHg**	84.5	78.25	90	79	72	81.75	<0.01
**TC mg/dL**	165.55	150.7	207.1	172.75	148.1	221.62	0.48
**LDL mg/dL**	86	69	105.75	88.5	64	119.25	0.86
**HDL mg/dL**	48.45	44.4	53.37	51.5	46.73	63.76	0.056
**non-HDL mg/dL**	113.7	98	155.75	118.75	95.95	159.74	0.79
**TG mg/dL**	169.45	109.23	204.55	152.55	117	193.5	0.87
**Glucose mg/dL**	161.9	143.1	197.38	134.4	117.9	170.78	<0.01
**ALT (U/I)**	27	21	49.5	30	26	43.75	0.96
**GGTP U/I**	39.5	30	53.75	38.5	28.25	53	0.90
**FIB-4**	1.5	1.18	1.98	1.38	1.15	1.67	<0.01

**Table 3:** Effect of glucagon-like peptide-1 receptor agonists (GLP-1RAs) on cytokine concentrations. Lp(a)—lipoprotein (a); MMP-9—matrix metalloproteinase-9; PTX3—pentraxin-3; SD—standard deviation; Q1—first quartile; Q3—third quartile.

	Study Group before Treatment			Study Group after Treatment			
	Mean	SD		Mean	SD		*p* Value
**Copeptin pg/mL**	190.44	66.67		172.83	52.35		*p* > 0.05
	**Median**	**Q1**	**Q3**	**Median**	**Q1**	**Q3**	
**Lp(a) mg/dL**	12.67	6.83	41.72	11.95	7.32	23.21	*p* < 0.05
**PTX3 pg/mL**	1288	1174.5	1381.5	1023.5	1008	1173	*p* < 0.001
**MMP-9 pg/mL**	294.15	256.48	312.7	277.35	245.28	303.63	*p* < 0.001

## Data Availability

Data are contained within the article.
